# Effect of implant tilt and trans-mucosal abutment angulation as influencing stress pattern: An FEA study

**DOI:** 10.6026/973206300213775

**Published:** 2025-10-31

**Authors:** Ambika Verma, Rajiv Kumar Gupta, Akshay Bhargava, Bharti Dua, Akanksha Sharma, Trisha Verma

**Affiliations:** 1 Department of Prosthodontics and Crown & Bridge, Santosh Dental College and Hospital, Santosh Deemed To Be University, Pratap Vihar, Ghaziabad, Uttar Pradesh, India

**Keywords:** Finite element analysis (FEA), dental implants, implant tilt, abutment angulation, stress distribution, maxillary arch, von mises stress, prosthetic components

## Abstract

The effect of implant tilt and transmucosal abutment angulation on stress distribution around tilted dental implants in a simulated maxillary arch using Finite Element Analysis (FEA). Eight 3D models with varying implant tilts (17°, 25°, 30°, 35°) and abutment angulations (17°, 30°) were analyzed under a 200 N vertical load. Matched angulations (e.g., 17°/17°, 30°/30°) showed more uniform and favorable stress distribution. Angular mismatches greater than 5° significantly increase stress in abutments and crestal bone. Implant tilts beyond 30° combined with mismatched abutments may compromise biomechanical stability and prosthetic success.

## Background:

Edentulism-complete tooth loss-affects oral function, aesthetics and self-confidence. Its prevalence is rising globally due to an aging population and limited access to dental care in some regions [1, 2]. Though preventive and restorative dentistry have advanced, edentulism remains a clinical challenge. Dental implant-supported prostheses have transformed rehabilitation for edentulous patients, offering enhanced mastication, aesthetics and long-term stability by preserving bone height and width [3]. Often termed the "third dentition," these restorations are now the standard of care for full-arch replacements, with the All-on-4 concept enabling immediate loading using a combination of straight and angled abutments [4]. Anatomical limitations-like buccal bone concavities or inadequate ridge width-often necessitate implant tilting, particularly in the maxilla. Angled transmucosal abutments (TMAs) help correct prosthetic alignment, but may alter force distribution, leading to biomechanical complications such as bone resorption or implant failure [5, 6]. Research indicates angled abutments can perform comparably to straight ones under certain conditions, but concerns remain regarding off-axis loading and stress on thin cortical plates. Finite Element Analysis (FEA) allows non-invasive, precise simulation of stress and strain on dental structures. Unlike photo elasticity or strain gauges, FEA provides detailed insight into the biomechanical behavior of implants under various conditions [7]. Studies show that posterior implant tilting reduces stress compared to vertical placement, while anterior implant tilt has minimal effect. Nevertheless, limited data exist on how TMA angulation interacts with different implant tilt angles. This study employs 3D FEA to evaluate how varying implant angulations (17°, 25°, 30° and 35°) combined with 17° and 30° TMAs affect stress distribution in the implant and adjacent bone under vertical and oblique loading. The study shows identify optimal implant-abutment configurations that reduce biomechanical stress and enhance prosthetic longevity [8]. Therefore, it is of interest to evaluate the effect of implant tilt and trans-mucosal abutment angulation as influencing the stress pattern.

## Methodology:

This in vitro Finite Element Method (FEM) study was conducted in the Department of Prosthodontics and Crown & Bridge at Santosh Dental College and Hospital, Delhi-NCR. The analysis was performed using ANSYS R17, robust FEM software known for its advanced meshing algorithms, accurate simulation of material properties and user-friendly interface. Its computational capabilities ensure enhanced modeling precision, result visualization and iterative refinement-critical for accurate biomechanical assessment. A three-dimensional maxillary model was constructed using Cone Beam Computed Tomography (CBCT) data obtained from a completely edentulous patient. The DICOM data were imported into SolidWorks 2014, where a solid anatomical model was generated. Cortical and cancellous bone layers were modeled distinctly, with cortical bone enveloping the cancellous core. A titanium dental implant (Adin, 3.75 mm x 11.5 mm) was modeled and digitally positioned in the anterior maxillary region (central incisor) at varying angulations of 17°, 25°, 30° and 35°, representing typical clinical scenarios involving anatomical limitations. For each implant angulation, two angled transmucosal abutments (TMA) of 17° and 30° were virtually attached, resulting in a total of eight experimental models. The specific combinations of implant and abutment angulations used in the study are summarized in Table 1 (see PDF), which outlines the geometric configuration of each model designed for comparative stress analysis. Each 3D model was then discretized through meshing in ANSYS R17 to achieve optimal accuracy in stress computation. The total number of elements and nodes for each model, along with their distribution among implant, abutment, cortical bone and cancellous bone components, are presented in Table 2 (see PDF). Fine meshing was employed in areas of high stress concentration, such as the implant-abutment interface and crestal bone region, to ensure precise stress mapping and computational stability. All materials used in the simulation were considered isotropic, homogeneous and linearly elastic, adhering to standard assumptions in finite element modeling of dental structures. The mechanical properties assigned to each material-Young's modulus, Poisson's ratio, tensile strength and density-are detailed in Table 3 (see PDF), ensuring accurate representation of their biomechanical behavior within the FEM environment. The bone-implant interface was assumed to be fully osseointegrated and a perfect fit was modeled between the implant, abutment and bone to eliminate confounding factors related to microgaps or interfacial slip. The superior surface of the maxillary model was constrained to simulate maxillary fixation, providing boundary stability. A vertical static occlusal load of 200 N was applied axially along the long axis of the implant-abutment assembly in each model, simulating average masticatory forces experienced by maxillary anterior teeth. Identical loading and boundary conditions were applied across all eight models for comparative consistency. A static structural analysis was performed using ANSYS R17 to determine von Mises stress distribution within the implant, abutment and surrounding cortical and cancellous bone structures. Color-mapped visual outputs were generated for each model, where red represented zones of maximum stress and blue indicated zones of minimal stress. This standardized setup ensured controlled, repeatable conditions to accurately compare stress patterns resulting from different implant and abutment angulations, as summarized through the configurations and parameters listed in Tables 1-3 (see PDF).

## Results:

The finite element analysis conducted across eight implant-abutment configurations revealed significant variations in biomechanical stress distribution depending on the degree of angular alignment or mismatch. Among all the models, Model 1-featuring a 17° implant tilt with a matching 17° transmucosal abutment (TMA)-demonstrated the most favorable biomechanical profile, with the lowest stress values in both the abutment (216.92 MPa) and implant (123.17 MPa), as well as moderate stress levels in cortical and cancellous bone (26.636 MPa and 4.2652 MPa, respectively). This indicated an efficient and nearly axial force transfer through the implant system. Conversely, Model 2 (implant at 17° with 30° abutment) exhibited the highest stress values in the abutment (544.84 MPa) and implant (235.01 MPa), confirming that a steep angular mismatch increases off-axis loading, shear forces and torque-particularly at the prosthetic interface. This misalignment also led to higher cortical bone stress (29.484 MPa), suggesting increased risk for marginal bone loss over time. Model 5, with a 30° implant and 17° abutment, further highlighted the detrimental effects of greater angular discrepancy. It registered the highest cortical bone stress (30.433 MPa), high abutment stress (467.80 MPa) and elevated cancellous bone stress (6.4 MPa), underscoring the compromised biomechanical environment resulting from a 13° mismatch. Interestingly, Model 4 (25° implant with 30° abutment), despite having a 5° mismatch, showed the lowest cortical bone stress (22.47 MPa), though it exhibited the highest cancellous bone stress (7.128 MPa) and high abutment loading (506.73 MPa) (Table 4 - see PDF, 5 - see PDF). These findings suggest that even small discrepancies can create localized stress concentrations depending on the angulation and anatomical site. Overall, the results emphasize that biomechanical performance is optimal when implant and abutment angulations are closely aligned. Greater mismatches lead to higher stress in prosthetic components and peri-implant bone, increasing the potential for clinical complications such as screw loosening, bone resorption, or implant failure. Proper prosthetic planning and angulation matching are therefore essential to ensure long-term success in implant-supported rehabilitations.

## Discussion:

Implant-supported prostheses are a cornerstone in modern prosthodontics, significantly improving function, aesthetics and patient satisfaction in edentulous cases. However, biomechanical complications remain a concern-particularly in the maxillary arch-due to anatomical limitations and variations in bone quality. This study used three-dimensional Finite Element Analysis (3D FEA) to evaluate the effect of implant tilt and transmucosal abutment (TMA) angulation on stress distribution. The results revealed that stress values increased as both implant tilt and abutment angulation increased. The combination of a 35° implant tilt with a 30° TMA produced the highest stress values in both implant components and the surrounding cortical bone. Douglass *et al.* (2002) highlighted the growing global demand for implant-supported prosthodontic rehabilitation driven by the aging population and the desire for functional and esthetic tooth replacement. This increasing clinical demand underscores the importance of biomechanical optimization of implant designs and angulations, as evaluated in the present finite element analysis. Ensuring optimal stress distribution in tilted implant configurations can directly influence the long-term success of prosthodontic treatment outcomes for edentulous or partially edentulous patients [9]. As noted by Petersen (2003), tooth loss remains a major global public health issue, with significant implications for function, nutrition and quality of life. Implant-based rehabilitation, particularly in anatomically challenging regions, plays a crucial role in addressing these concerns. The present study contributes to this global objective by biomechanically evaluating the effects of implant tilt and abutment angulation-parameters that can improve implant placement feasibility while maintaining favorable stress distribution and structural integrity [10]. Consistent with the observations of Soni *et al.* (2020), the present study reinforces the importance of implant angulation and abutment configuration in determining stress distribution patterns within the peri-implant bone. Variations in implant tilt and transmucosal abutment angulation can either concentrate or dissipate stresses in cortical and cancellous regions. By analyzing these parameters through finite element modeling, the current research extends previous findings by offering a comparative biomechanical perspective on angled abutments and tilted implants in anterior maxillary rehabilitation [11]. Stress levels were noticeably higher in models using 30° TMAs than in those with 17° abutments, especially when paired with highly tilted implants. Excessive angulation can create off-axis loading and concentrated force transmission, which may compromise osseointegration or lead to mechanical failures [12]. The findings of Zarb and Schmitt (1993) underscored the critical role of achieving and maintaining osseointegration for long-term implant success. Building on this principle, the current finite element analysis explores how variations in implant tilt and transmucosal abutment angulation may influence stress concentration at the bone-implant interface. Since excessive stress can compromise osseointegration and lead to marginal bone loss, optimizing implant-abutment geometry, as analyzed in this study, is essential for enhancing the long-term biomechanical stability and clinical success of implant-supported restorations [13]. The study demonstrates that varying angle correction designs for inclined implants in the All-On-Four protocol significantly influence stress distribution, highlighting the importance of precise abutment angulation in minimizing biomechanical complications [14]. It remains the gold standard in simulating and evaluating biomechanical conditions in implant dentistry. In summary, the combination of a moderately tilted implant (25°) with a conservative abutment angulation (17°) was the most biomechanically favorable. This configuration effectively minimized stress on implants and peri-implant bone, promoting long-term stability and prosthetic success.

## Conclusion:

We show that both implant tilt and abutment angulation alignment significantly affect stress distribution in implant-supported prostheses. Matched angulations (≤5° difference) result in more favorable biomechanical outcomes, while excessive tilt (>30°) and mismatches increase stress concentrations, especially in the abutment collar region. Precise angulation control and prosthetic planning are essential for minimizing mechanical complications and ensuring long-term implant success.
